# Supplementary figures and images for: Engineering High-Yield Biopolymer Secretion Creates an Extracellular Protein Matrix for Living Materials
Source: mSystems. 2021 Mar 23;6(2):e00903-20. doi: 10.1128/mSystems.00903-20 (PMC8546985; doi:10.1128/mSystems.00903-20)

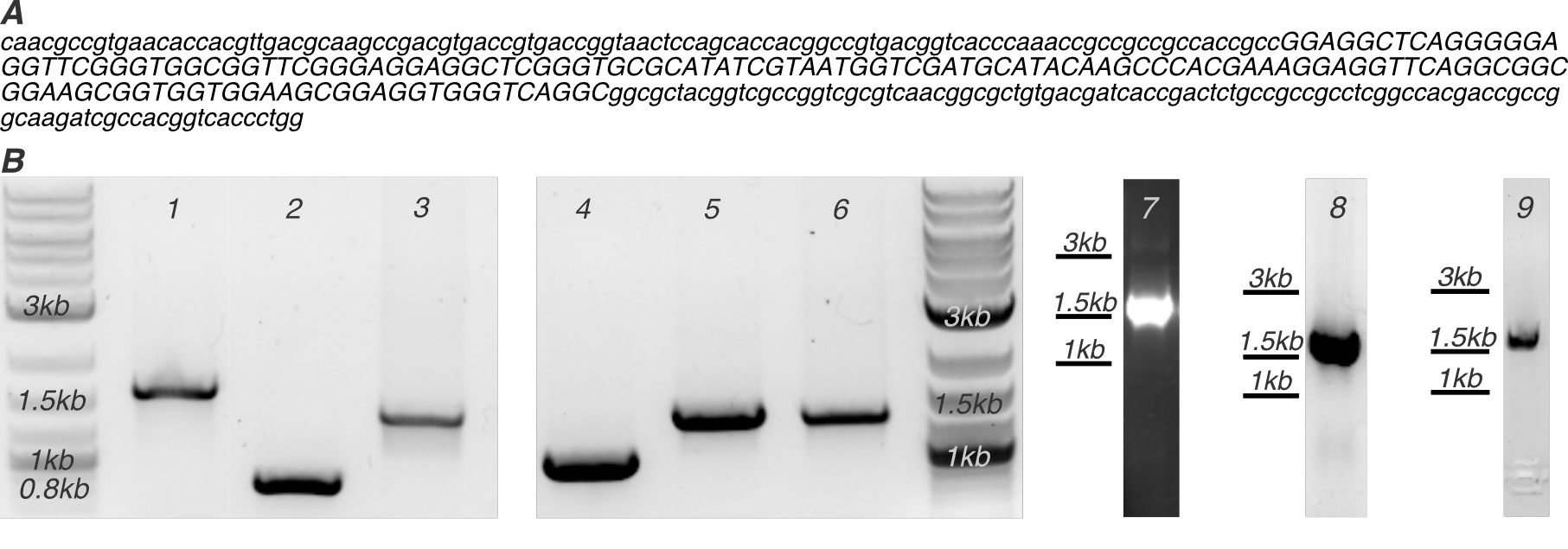

Supplement: FIG S1 [file msystems.00903-20-sf001.tif]

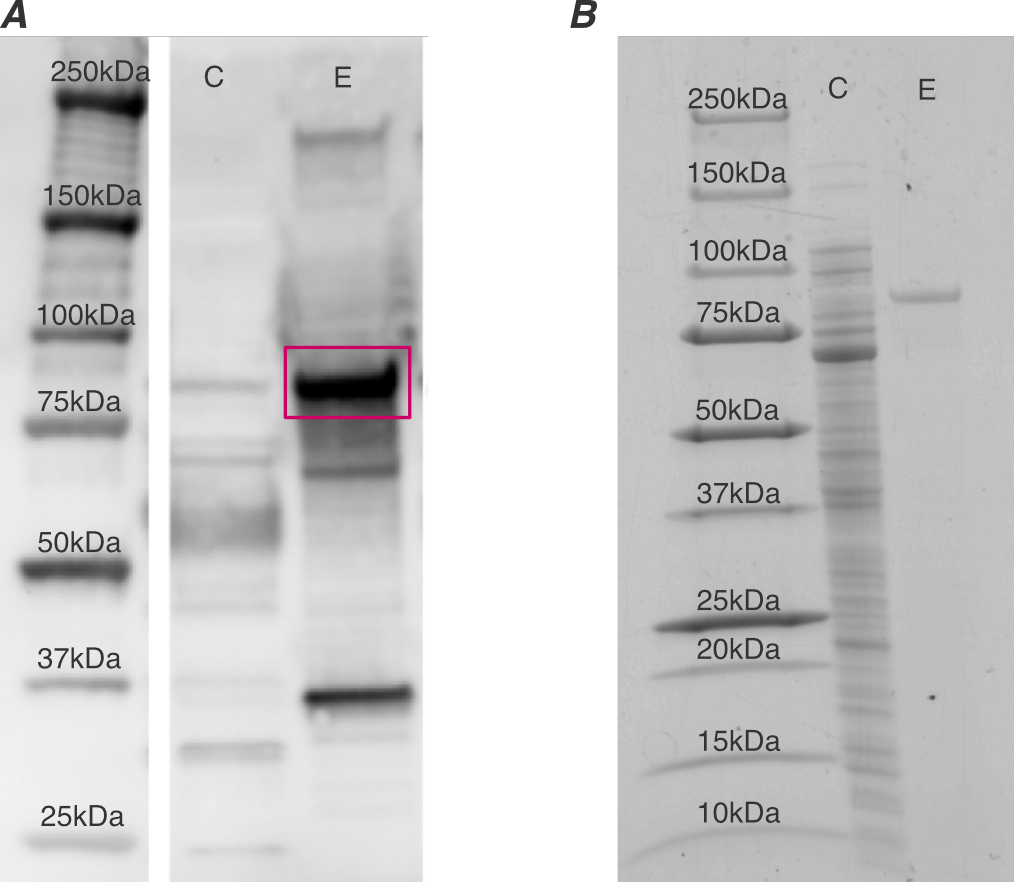

Supplement: FIG S2 [file msystems.00903-20-sf002.tif]

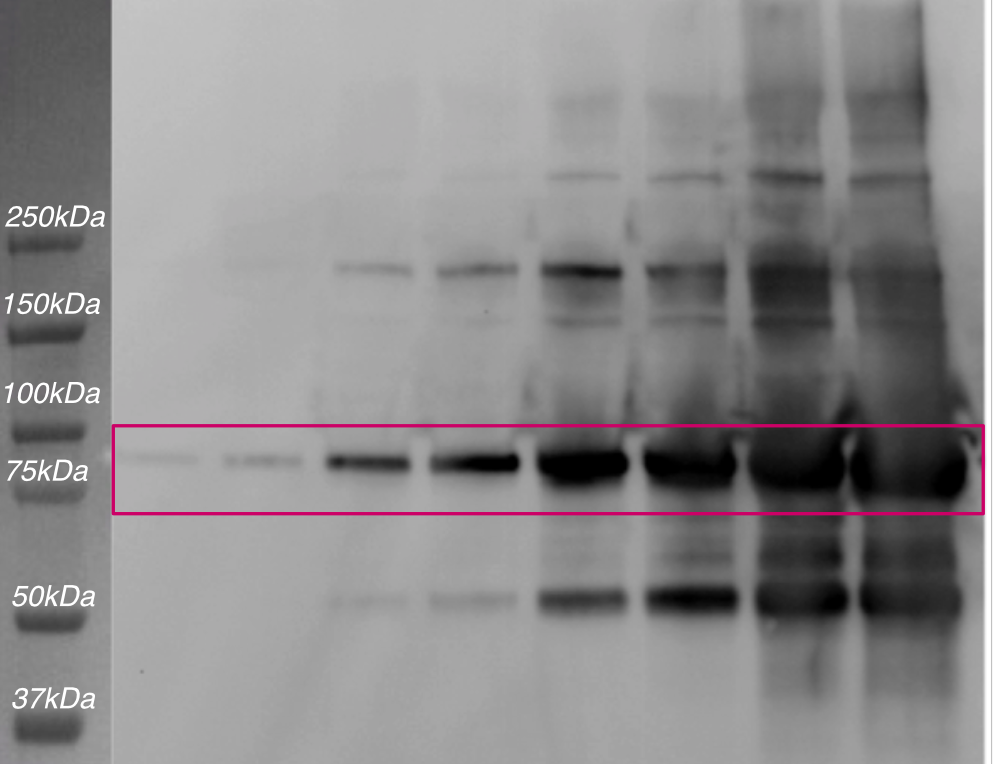

Supplement: FIG S3 [file msystems.00903-20-sf003.tif]

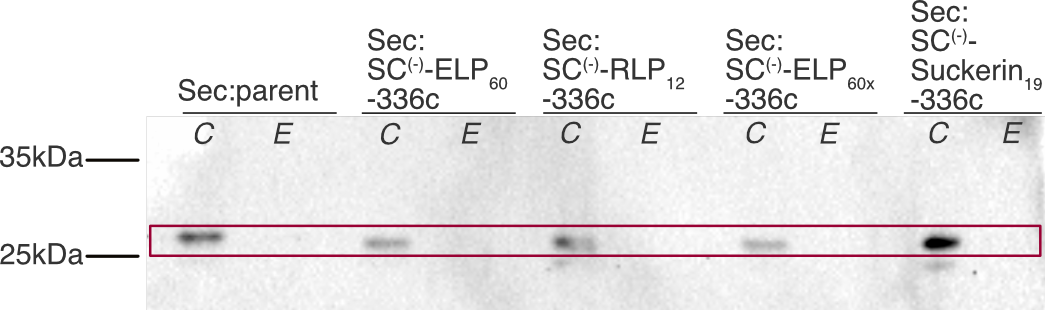

Supplement: FIG S5 [file msystems.00903-20-sf005.tif]

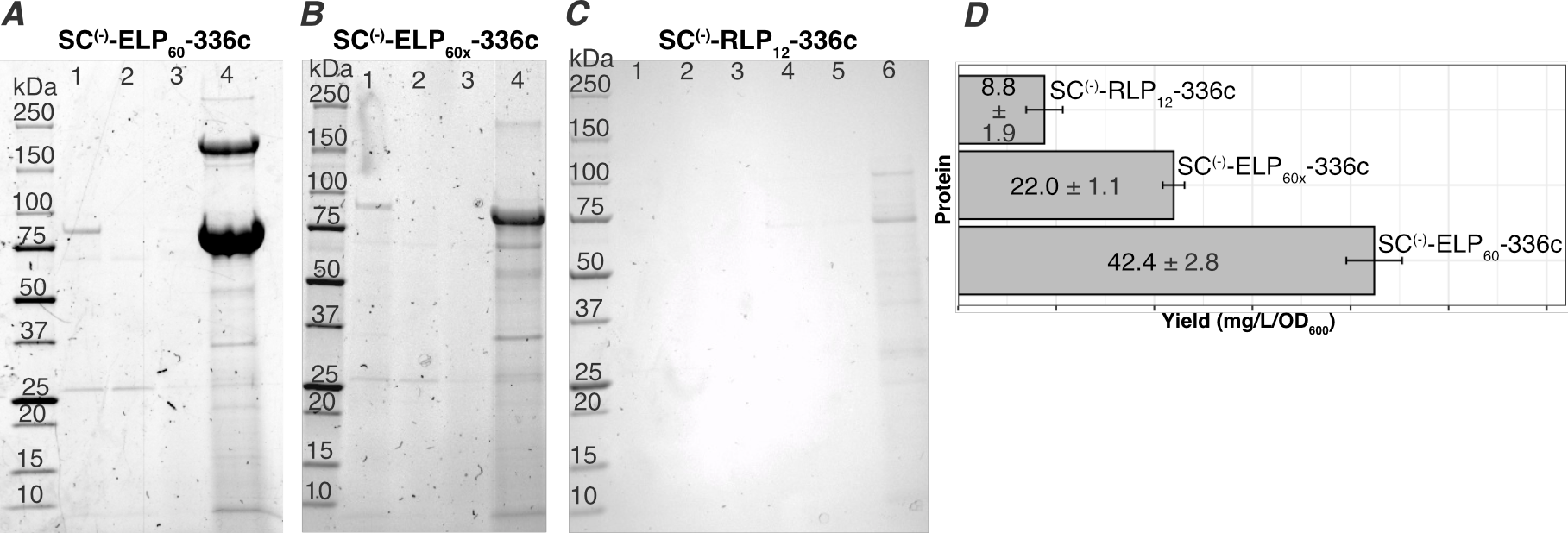

Supplement: FIG S4 [file msystems.00903-20-sf004.tif]

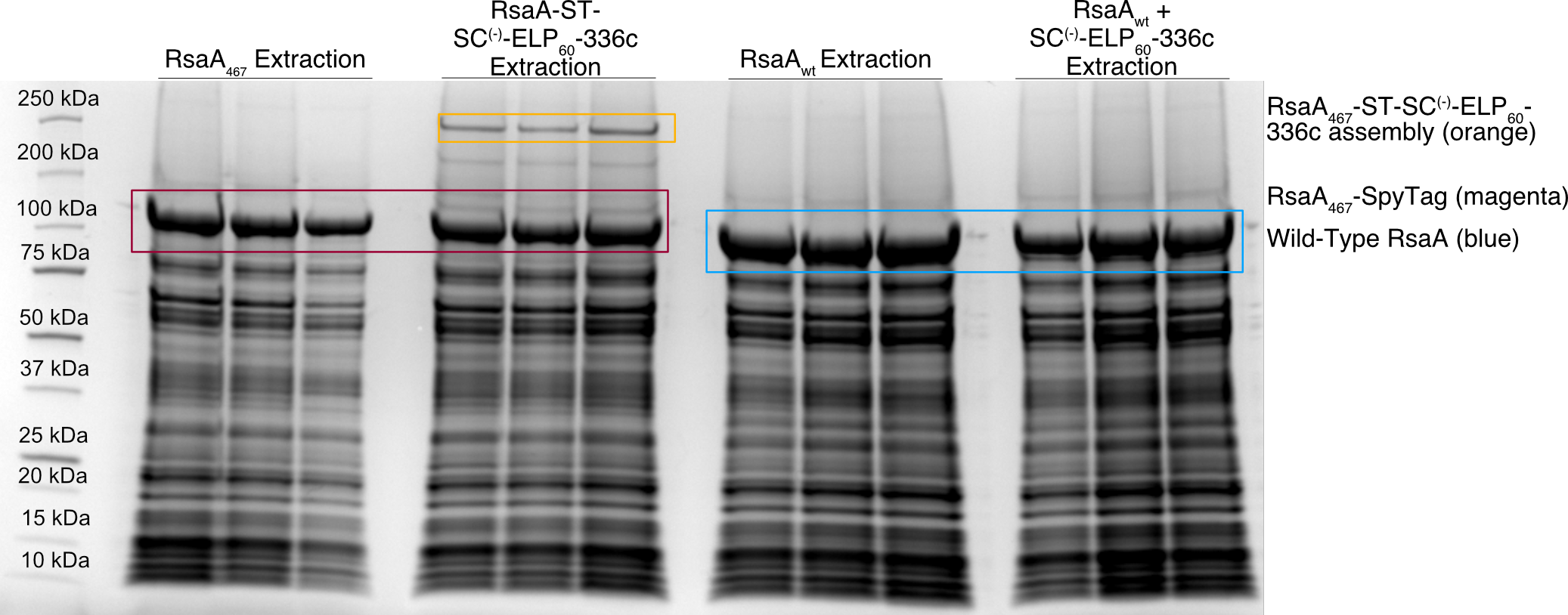

Supplement: FIG S6 [file msystems.00903-20-sf006.tif]
