# Supplementary material for: Engineering High-Yield Biopolymer Secretion Creates an Extracellular Protein Matrix for Living Materials
Source: mSystems. 2021 Mar 23;6(2):e00903-20. doi: 10.1128/mSystems.00903-20 (PMC8546985; doi:10.1128/mSystems.00903-20)
Supplement: TABLE S1 [file msystems.00903-20-st001.docx]

###

| **Name** | **Characteristics** | **Plasmid** | **Strain background** | **Source** |
| --- | --- | --- | --- | --- |
| MFm092 Disp:RsaA_wt_ | *C. crescentus* CB15 Δ*sapA*::Pxyl-*gfpmut3* | None | CB15 | This study |
| MFm109 Disp:RsaA_467_-ST | *C. crescentus* CB15 Δ*sapA*::Pxyl-*gfpmut3 rsaA_467_-spytag* | None | CB15 | This study |
| Mfm126  Sec:parent | *C. crescentus* CB15N Δ*sapA*::Pxyl-*mKate2* | None | CB15N | This study |
| Mfm 127  Sec:SC-336c | *C. crescentus* CB15N Δ*sapA*::pXyl-*mKate2* Δ*rsaA*::PrsaA-*spycatcher-336c* | None | CB15N | This study |
| Mfm 142 Sec:336c | *C. crescentus* CB15N Δ*sapA*::pXyl-*mKate2* Δ*rsaA*::PrsaA-*336c* | None | CB15N | This study |
| Mfm 144 Sec: SC^(-)^-ELP_60_-336c | *C. crescentus* CB15N Δ*sapA*::Pxyl-*mKate2* Δ*rsaA*::PrsaA-*sc^(-)^-elp_60_-336c* | None | CB15N | This study |
| Mfm 145 | B5BAC p336c-*spycatcher* | p336c-SpyCatcher | B5BAC | This study |
| Mfm 149 Sec:SC^(-)^-RLP_12_-336c | *C. crescentus* CB15N Δ*sapA*::Pxyl-*mKate2* Δ*rsaA*::PrsaA-*sc^(-)^-rlp_12_-336c* | None | CB15N | This study |
| Mfm151 Sec:SC^(-)^-Suckerin_19_-336c | *C. crescentus* CB15N Δ*sapA::*Pxyl-*mKate2* Δ*rsaA*::PrsaA-*sc^(-)^-suckerin_19_-336c* | None | CB15N | This study |
| Mfm 152 Sec:ELP_60_-336c | *C. crescentus* CB15N Δ*sapA*::Pxyl-*mKate2* Δ*rsaA*::PrsaA-*elp_60_-336c* | None | CB15N | This study |
| Mfm 159 Sec:SC-ELP_60_-336c | *C. crescentus* CB15N Δ*sapA*::Pxyl-*mKate2* Δ*rsaA*::PrsaA-*spycatcher-elp_60_-336c* | None | CB15N | This study |
| Mfm 161 Sec:SC^(-)^-ELP_60x_-336c | *C. crescentus* CB15N Δ*sapA::*Pxyl-*mKate2* Δ*rsaA*::PrsaA-*spycatcher^(-)^-elp_60x_-336c* | None | CB15N | This study |
| Mfe 939 WM3064 | DAP auxotroph *E. coli* strain for conjugation | None | WM3064 | W. Metcalf/ UIUC |

### 
