# Supplementary material for: Engineering High-Yield Biopolymer Secretion Creates an Extracellular Protein Matrix for Living Materials
Source: mSystems. 2021 Mar 23;6(2):e00903-20. doi: 10.1128/mSystems.00903-20 (PMC8546985; doi:10.1128/mSystems.00903-20)
Supplement: TABLE S3 [file msystems.00903-20-st003.docx]

| **Protein/Domain** | **Isoelectric point (pI)** |
| --- | --- |
| 336c | 3.83 |
| SC | 4.46 |
| SC-336c | 4.14 |
| SC-ELP_60_-336C | 4.16 |
| ELP_60_-336c | 3.99 |
| SC^(-)^ | 3.98 |
| SC^(-)^-ELP_60_-336c | 3.97 |
| SC^(-)^-RLP_12_-336c | 4.33 |
| SC^(-)^-ELP_60x_-336c | 5.09 |
| ELP_60_ | 5.52 |
| RLP_12_ | 9.91 |
| ELP_60x_ | 10.7 |
| Suckerin_19_ | 8.33 |

### 
