# Supplementary material for: Engineering High-Yield Biopolymer Secretion Creates an Extracellular Protein Matrix for Living Materials
Source: mSystems. 2021 Mar 23;6(2):e00903-20. doi: 10.1128/mSystems.00903-20 (PMC8546985; doi:10.1128/mSystems.00903-20)
Supplement: TEXT S1 [file msystems.00903-20-s0001.docx]

**SUPPLEMENTAL METHODS**

### **Plasmid construction.**

For the construction of the background strains (*C. crescentus* CB15N Δ*sapA*::Pxyl-*mkate2* and *C. crescentus* CB15 Δ*sapA*::Pxyl-*GFPmut3*), a pNPTS138 integration plasmid was used to remove the S-layer protease gene (*sapA*, *CCNA_00783*) and replace it with a gene encoding a mKate2 or GFPmut2 fluorescent protein under a xylose-induction promoter. We PCR amplified 800 base pairs of the upstream and downstream regions of *sapA* using primers sapA_US and sapA_DS (Table 2) from genomic DNA purified from *C. crescentus* CB15N cells using DNEasy kit (Qiagen). The pXyl-GFPmut3 sequence (Table 2) with homology regions for assembly was synthesized by Integrated DNA Technologies. The pNPTS138 backbone was digested with HindIII-HF and SphI-HF restriction enzymes in CutSmart buffer (New England Biolabs). Subsequently, the sequence verified pNPTS138-sapA-GFP plasmid was amplified with PCR to remove *gfp* with pNPTS_sapA_F and pNPTS_sapA_R primers. The mKate2 sequence was PCR amplified with homology regions using pNPTS_mKate2-F and pNPTS_mKate2-R primers (Table 2) from the pXGFPC-2 Plac::mKate2 plasmid [(75)](https://paperpile.com/c/fe3J28/dl1wa).

The p336c-SC plasmid is not used in the study except as a source plasmid for subsequent pNPTS138 construction. It was constructed from a synthetic *spycatcher* gene (Table 2, Integrated DNA Technologies) and codon-optimized for *C. crescentus.* The synthetic gene was PCR amplified with SC_336_F and SC_336_R primers and the p336c backbone plasmid (courtesy of the Smit Lab, UCB) linearized with the 336c_start_F and 336c_start_R primers.

To construct the pNPTS138-SC-336c integration plasmid, pNPTS138 was digested with NheI and HindIII-HF restriction enzymes in CutSmart buffer (New England BioLabs) for 3 hours at 37°C. The *spycatcher-336c* sequence was PCR amplified from the previously constructed p336c-SC plasmid using primers FLAG-SC-F and 336-pNPTS_R. The upstream sequence of *rsaA* (*CCNA_01059*) was amplified from the genomic DNA of *C. crescentus* CB15, and purified with a DNEasy kit (Qiagen) using US_rsaA_F and US_rsaA_R primers (Table 2). Subsequently, pNPTS138-336c was created from pNPTS138-SC-336c using the Q5 mutagenesis kit (New England BioLabs) with primers pNPTS-336_F and pNPTS_USrsaA_R. *sc^(-)^-hydrogel* (*sc^(-)^-elp_60_, sc^(-)^-rlp_12_,* or *sc^(-)^-suckerin_19_*) fusion gene sequences were synthesized and inserted into the pUC57 plasmid (Genscript). These were digested with BamHI-HF and either ApoI-HF or EcoRI-HF restriction enzymes in CutSmart buffer (New England BioLabs) for 4-5 hours at 37°C. The digests were then assembled with a pNPTS138-SC-336c plasmid linearized to remove *sc* with the primer pairs 336-scSC-ELP60_F with pNPTS_superSC_R2, 336-scSC-RLP-F with pNPTS_superSC_R or 336-scSC-Suckerin-F with pNPTS_superSC_R. pNPTS138-SC^(-)^-ELP60_x_-336c was constructed in its entirety by Genscript. pNPTS138-ELP_60_-336c was constructed from the pNPTS138-336c plasmid linearized with primers pNPTS_ELP60_F and pNPTS_ELP60_R and and *elp_60_* digested with Apo1-HF and BamHI-HF in CutSmart buffer for 5 hours at 37°C from the pUC57 source plasmid. pNPTS138-SC-ELP_60_-336c was constructed from pNPTS138-336c linearized with primers 336c-scSC-ELP60-F and pNPTS138-336c_R, *spycatcher* amplified from p336c-SC with FLAG-SC_F and SC-STREP_R using Phusion High-Fidelity PCR Master Mix with HF Buffer, and *elp_60_* digested from p336c-ELP_60_ with ApoI-HF and BamHI-HF at 37°C for 6 hours.

All PCR and restriction digest fragments were visualized on 1% agarose gels with 1x SYBR Safe DNA Gel stain (ThermoFisher Scientific). DNA bands were excised and purified with QX1 solubilization buffer (Qiagen) and the DNA Clean & Concentrate kit (Zymo Research) according to the manufacturer's guidelines. All PCR reactions were performed using Q5 High Fidelity 2x Master Mix (New England BioLabs) according to the manufacturer's guidelines. Gibson Assembly was carried out using HiFi Gibson Assembly 2x Master Mix (New England BioLabs) for 1 hour at 50°C. 5 µl of each Gibson assembly reaction was transformed into NEB 5-alpha chemically competent cells (New England Biolabs) and plated on LB supplemented with kanamycin to select for the pNPTS138 plasmids. Successful assemblies were confirmed by Sanger sequencing at the UC Berkeley DNA Sequencing Facility (Berkeley, CA, USA).
