# Supplementary material for: Engineering High-Yield Biopolymer Secretion Creates an Extracellular Protein Matrix for Living Materials
Source: mSystems. 2021 Mar 23;6(2):e00903-20. doi: 10.1128/mSystems.00903-20 (PMC8546985; doi:10.1128/mSystems.00903-20)
Supplement: TABLE S2 [file msystems.00903-20-st002.docx]

###

| **Plasmids** | | |
| --- | --- | --- |
| **Name** | **Characteristic** | **Source** |
| puc57_SuperchargedSC-ELP_60_ | Storage plasmid for *spycatcher^(-)^-elp_60_* gene. | GenScript |
| puc57_SuperchargedSC-RLP_12_ | Storage plasmid for *spycatcher^(-)^-rlp_12_* gene. | GenScript |
| puc57_SuperchargedSC-Suckerin_19_ | Storage plasmid for *spycatcher^(-)^-suckerin_19_* gene. | GenScript |
| p336c | Plasmid for expression of proteins via the *C. crescentus* B5BAC T1SS. Contains the 336c secretion signal. | John Smit, University of British Columbia |
| p336c-SpyCatcher | Plasmid expression of original SpyCatcher with 336c signal through T1SS. | This study |
| p336c-ELP_60_ | Plasmid expression of *elp_60_* with *336c* signal through T1SS. | This study |
| pXGFPC-2 Plac::mKate2 | Contains *mKate2* sequence. | Persat[(75)](https://paperpile.com/c/fe3J28/dl1wa) |
| pNPTS138 | Plasmid for genomic insertion using two-step recombination with SacB counterselection. | M. R. K. Alley |
| pNPTS138-ΔsapA::Pxyl-mKate2 | Genomic insertion of *mKate2* under xylose induction in place of *sapA* gene. | This study |
| pNPTS138-SC-336c | Genomic insertion of *spycatcher* in place of *rsaA_1-690_.* | This study |
| pNPTS138-336c | Genomic removal of *rsaA_1-690_* leaving *336c* secretion signal sequence. | This study |
| pNPTS138-ELP_60_-336c | Genomic insertion of *elp_60_* in place of *rsaA_1-690_.* | This study |
| pNPTS138-SC-ELP_60_-336c | Genomic insertion of *spycatcher-elp_60_* in place of *rsaA_1-690_.* | This study |
| pNPTS138-superchargedSC-336c | Genomic insertion of *spycatcher^(-)^* in place of *rsaA_1-690_.* | This study |
| pNPTS138-superchargedSC-ELP_60_-336c | Genomic insertion of *spycatcher^(-)^-elp_60_* in place of *rsaA_1-690_.* | This study |
| pNPTS138-superchargedSC-RLP_12_-336c | Genomic insertion of *spycatcher^(-)^-rlp_12_* in place of *rsaA_1-690_.* | This study |
| pNPTS138-superchargedSC-Suckerin_19_-336c | Genomic insertion of *spycatcher^(-)^-suckerin_19_* in place of *rsaA_1-690_.* | This study |
| pNPTS138-superchargedSC-ELP60x-336c | Genomic insertion of *supercharged spycatcher-elp_60x_* in place of *rsaA_1-690_.* | This study |
| **Primers** | | |
| **Name** | **Characteristics** | **Sequence** |
| pNPTS_sapA_R | To amplify pNPTS138-sapA-eGFP removing *eGFP* | CATGTCGTCTCCCCAAAACTCGAGCGTCTGAAGC |
| pNPTS_sapA_F | To amplify pNPTS138-sapA-eGFP, removing *eGFP* | CCGTTCGAAGGGCGCGGCGAC |
| pNPTS-mKate2_F | To amplify *mKate2* with overlaps to pNPTS138-sapA | gacgctcgagttttggggagacgacATGGTGAGCGAGCTGATTAAGGAGAACATG |
| pNPTS-mKate2_R | To amplify *mKate2* with overlaps to pNPTS138-sapA | gtcgccgcgcccttcgaacggtcatctGTGCCCCAGTTTGCTAGGGAGGTC |
| 336-pNPTS_R | To obtain *sc-336c* from p336c-SC. | cgcgtcacggccgaagctagcGTCGCAGCAGCGCCCAGGGTG |
| FLAG-SC-F | To amplify *sc-336c* from p336c-SC. | gactacaaggacgacgatgacaagGCGATGGTGGATACGCTCTCCGGC |
| US_rsaA_F | To amplify upstream *rsaA* from CB15 genome. | caattgaagccggctggcgccaagcttCTCAGGCCGCGATCAGTGCCGAC |
| US_rsaA_R | To amplify upstream *rsaA* from CB15 genome. | catcgccttgtcatcgtcgtccttgtagtcCATGAGGATTGTCTCCCAAAAAAAATCCCACACCC |
| 336-scSC-F | To amplify pNPTS138-SC-336c with overlap to *spycatcher^(-)^*. Includes *336c* sequence. Excises original *spycatcher* gene | cacatcgacgggggctgcggcaaatttGCTGACCCGGCCTTCGGCGGC |
| pNPTS_superSC_R | To amplify pNPTS138-SC-336c with overlap to *spycatcher^(-)^.* | gcgtgtccaccatggccatgaattcCTTGTCATCGTCGTCCTTGTAGTCCATGAGG |
| pNPTS_superSC_R2 | To amplify pNPTS138-SC-336c with overlap to *spycatcher^(-)^.* | CGGACAGCGTGTCCACCATGGCCATgaattcCTTGTCATCGTCGTCCTTGTAGTCCATGAGG |
| 336-scSC-ELP60_F | Amplify pNPTS138-336c with overlaps to *sc-elp_60_-336c*. | caggcggctcgggcggatccGCTGACCCGGCCTTCGGCGG |
| pNPTS138-336c_R | Amplify pNPTS138-336c with overlaps to *sc-elp_60_-336c.* | CTTGTCATCGTCGTCCTTGTAGTCCATGAGGATTGTCTCCC |
| 336-scSC-RLP F | To amplify pNPTS138-SC-336c with overlap to *rlp_12_*. Includes *336c* sequence. Excises original *spycatcher* gene. | cctggggggcggctcgggcggatccGCTGACCCGGCCTTCGGCGG |
| 336-scSC-Suckerin F | To amplify pNPTS138-SC-336c with overlap to *suckerin_19_*. Includes *336c* sequence. Excises original *spycatcher* gene. | gctccacggcggctcgggcggatccGCTGACCCGGCCTTCGGCGG |
| pNPTS_USrsaA_R | To amplify pNPTS138-SC-336c with no overlap. Includes *336c* sequence. Excises original *spycatcher* gene. | CTTGTCATCGTCGTCCTTGTAGTCCATGAGGATTGTCTCCC |
| pNPTS-336_F | To amplify pNPTS138-SC-336c with no overlap. Includes *336c* sequence. Excises original *spycatcher* gene. | GCTGACCCGGCCTTCGGCGGC |
| pNPTS-336_R | To amplify pNPTS138-SC-336c with no overlap. Includes *336c* sequence. Excises original *spycatcher* gene. | CTTTTCAAACTGCGGGTGGGACCAaaatttGCC |
| FLAG-SC_F | Amplify *spycatcher* with overlaps to backbone and *strep-elp_60_*. | gactacaaggacgacgatgacaagGCGATGGTGGATACGCTCTCCGGC |
| SC-STREP_R | Amplify *spycatcher* with overlaps to backbone and *strep-elp_60_*. | gcgggtgggaccaaaatttGCCGCTCCCGCCGTCGATATG |
| pNPTS_ELP60_F | To amplify pNPTS138-336c with overlap to *elp_60_*. | CCAGGCGGCTCGGGCggatccGCTGACCCGGCCTTCGGCGG |
| pNPTS_ELP60_R | To amplify pNPTS138-336c with overlap to *elp_60_*. | AACTCCCTTTTCAAACTGCGGGTGGGACCAaaatttCTTGTCATCGTCGTCCTTGTAGTCCATGAGGATTG |
| SacB-F | Colony PCR primer to confirm removal of *SacB* from genome. | GGAAGCTCGGCGCAAACGTTGATTG |
| SacB-R | Colony PCR primer to confirm removal of *SacB* from genome. | CCACATCGTCTTTGCATTAGCCGGAGATCC |
| KI-336c_R | Colony PCR primer, to confirm  removal of *rsaA*, leaving only *flag-336c*. | CAGCCTTGTCATCGTCGTCCTTGTAGTCC |
| KI_RLP12_R | Colony PCR primer, to confirm  knock-in of *sc^(-)^-rlp_12_* genes after PrsaA and before *336c*. | GAGTCGGACGGACGCCCGCCATTC |
| KI_Suckerin_R | Colony PCR primer, to confirm  knock-in of *sc^(-)^-suckerin_19_* genes after PrsaA and before *336c*. | CCAGGCCGTAGCCGCCGTAGAGG |
| KI_SC_F | Colony PCR primer, knock-in confirmation of *spycatcher*. | GACGAACCAGGGTTCGTTCTCGTCGC |
| ColonyPCR_336c_R | Colony PCR primer, knock-in confirmation of 336c. | GATCGACTTGGCCGAGGTGGCTTGCA |
| ColonyPCR_ELP60_R | Colony PCR primer, knock-in confirmation of *sc^(-)^-elp_60_* and *elp_60_*. | ACACCTGCTCCGGGAACTCCCCCA |
| ColonyPCR_ELP60X_R | Colony PCR primer, knock-in confirmation of *sc^(-)^-elp_60x_*. | CCCTTGCTGGCCTGGAACTCCTA |
| SC_336_F | To amplify the *ggsg_spycatcher_CB* gene block | caatttcacacaggaaacagctatgGCGATGGTGGATACGCTCTCCGGCCTGTCG |
| SC_336_R | To amplify the *ggsg_spycatcher_CB* gene block | cgggaattcgtaatcatggtGCCGCTCCCGCCGTCGATATGGGCGTCGCCCTTGGTCGCC |
| 336C_start_F | To linearize the p336c plasmid. | ACCATGATTACGAATTCCCGGGGATCC |
| 336C_start_R | To linearize the p336c plasmid. | CATAGCTGTTTCCTGTGTGAAATTGTTATCCGC |
| **Synthesized gene blocks** | | |
| **Name** | **Characteristics** | **Sequence** |
| GGSG_SpyCatcher_CB | SpyCatcher with GGSG linker sequence codon optimized for *C. crescentus*. Synthesized by Integrated DNA Technologies | GCGATGGTGGATACGCTCTCCGGCCTGTCGTCGGAGCAAGGGCAGTCGGGGGATATGACCATCGAGGAGGATTCGGCCACCCACATCAAGTTCTCCAAGCGTGATGAAGACGGGAAGGAACTCGCCGGGGCCACGATGGAGCTCCGCGACTCGTCCGGGAAGACCATCTCCACCTGGATCTCGGATGGGCAAGTGAAGGACTTTTATCTCTACCCCGGGAAGTATACGTTTGTCGAGACGGCGGCCCCCGATGGGTACGAGGTGGCGACCGCGATCACGTTTACCGTCAATGAACAGGGGCAGGTCACCGTGAACGGCAAGGCGACCAAGGGCGACGCCCATATCGACGGCGGGAGCGGC |
| pXyl-GFPmut3 | pXyl-GFPmut3 sequence codon optimized for *C. crescentus.* Synthesized by Integrated DNA Technologies | ATACTCCTTTCAGGTGAGTGGAGCGCGTCGCTGCAGCCAGCCGTGGTCGGGCAGCAGGTAGAAGGCGCCCTCGTCCTGATCCTCGCCCGAAACCTCCAGCCCCCGGTCGATGGCTTCGACGACATAGCCGGCCGCGCGGCAGGTGTCGGTGAGCGCGGCCAGCAGGGCGGCTTCCTGGTCAGGGGTCAGGTCGGTCATGGGCAAGAGGTCCAGGTCGTGGTTTGTCGGCGGCTTCTAGCATGGACCGCCCGCGCCCGTGAGGCCGAGGATTTCGCGCTGGTCAGACAACCTACTTGCCGTCCCCACATGTTAGCGCTACCAAGTGCCGACGAACGCGCGCCGCCGACGGTGTCGGCGCTTCAGACGCTCGAGTTTTGGGGAGACGACATGCGGAAGGGCGAAGAACTCTTTACCGGGGTGGTCCCGATCCTCGTGGAGCTCGATGGCGATGTCAATGGGCACAAGTTCTCCGTCTCGGGCGAAGGGGAGGGGGATGCCACGTATGGCAAGCTCACGCTGAAGTTCATCTGCACCACCGGCAAGCTCCCCGTGCCCTGGCCCACCCTCGTCACGACGTTCGGCTATGGCGTGCAATGCTTTGCCCGTTATCCGGATCATATGAAGCGCCATGATTTTTTTAAGAGCGCCATGCCCGAAGGGTATGTGCAGGAACGGACCATCTTCTTCAAGGACGACGGGAATTACAAGACCCGGGCGGAGGTGAAGTTCGAGGGGGATACGCTGGTGAATCGCATCGAGCTCAAGGGGATCGACTTCAAGGAGGACGGCAACATCCTGGGGCACAAGCTCGAATATAACTATAACTCGCACAATGTCTATATCATGGCCGATAAGCAAAAGAACGGGATCAAGGTGAACTTCAAGATCCGGCATAACATCGAGGACGGGTCGGTGCAGCTCGCCGACCATTACCAGCAGAATACGCCCATCGGGGATGGGCCGGTGCTGCTGCCCGACAATCACTATCTCAGCACGCAATCCGCGCTGTCGAAGGACCCGAATGAAAAGCGCGACCATATGGTCCTGCTGGAGTTTGTCACGGCCGCCGGCATCACGCATGGCATGGATGAACTGTATAAGTAACCGTTCGAAGGGCGCGGCGACAAAGGTCCA |
